# Biologics for the treatment of chronic rhinosinusitis with nasal polyps - state of the art

**DOI:** 10.1016/j.waojou.2019.100050

**Published:** 2019-08-09

**Authors:** Lei Ren, Nan Zhang, Luo Zhang, Claus Bachert

**Affiliations:** a Upper Airways Research Laboratory, Ghent University, Ghent, Belgium; b Department of Allergy, Beijing Tongren Hospital, Capital Medical University, Beijing, China; c Department of Otolaryngology Head and Neck Surgery, Beijing Tongren Hospital, Capital Medical University, Beijing, China; d Beijing Key Laboratory of Nasal Diseases, Beijing Institute of Otolaryngology, Beijing, China; e Division of ENT Diseases, CLINTEC, Karolinska Institute, Stockholm, Sweden

**Keywords:** Nasal polyps, Biologics, Mepolizumab, Omalizumab, Dupilumab

## Abstract

Chronic rhinosinusitis with nasal polyps (CRSwNP) is a complex upper airway disease affecting up to 11% of the population of Western Europe. In these western countries, 85% of the CRSwNP disease reveals a type 2 inflammatory pattern. In the last 15 years, several randomized double-blind studies on monoclonal antibodies in CRSwNP were performed. These studies demonstrated for the first time that biologics targeting type 2 immune reactions might be successful in nasal polyps. The target proteins, interleukin (IL)-4, IL-5, IL-13, and IgE were previously identified as key mediators in studies using nasal polyp tissues to measure and to interact in ex-vivo settings. No biomarkers have been identified to predict response to a specific biologic or to monitor treatment success. These studies were characterized by small numbers of patients and heterogeneous populations. They did, however, pave the way for currently performed and analyzed phase 3 studies, which will possibly lead to the registration of the first biologic drug with the indication CRSwNP. The studies already provide indications on the effects to be expected from those biologics; the results of phase-3 studies in larger populations will be decisive for the indications, patient selection, and finally the stopping rules for those drugs in subjects with severe nasal polyps, in whom the current standard of care including topical and oral glucocorticosteroids, antibiotics and surgical procedures failed to control the disease. We may expect that those biologics will open new perspectives for those patients with severe polyposis with, but also independent of asthma, allowing to avoid the possible adverse events resulting from systemic glucocorticosteroids and surgery.

## Abbreviationns

IL: Interleukin
IgE: Immunoglobulin E
CRS: Chronic rhinosinusitis
CRSwNP: Chronic rhinosinusitis with nasal polyps
RCT: Randomized controlled trial
GCS: Glucocorticosteroid
INCS: Intranasal corticosteroid spray
MFNS: Mometasone furoate nasal spray
FANS: Flixonase aqueous nasal spray
TPS: Total nasal polyp score
CT: Computed tomography
LOCF: Last observation carried forwad
UPSIT: University of Pennsylvania Smell Identification Test
VAS: Visual Analog Scale
SNOT-22: 22-item SinoNasal Outcome Test
SF-36: Short-Form health questionnaire
RSOM-31: 31-Item Rhinosinusitis Outcome Measuring Instrument
ACQ5: 5-item Asthma Control Questionnaire
AQLQ: Asthma Quality of Life Questionnaire
ESS: Endoscopic sinus surgery
PnIF: Peak nasal inspiratory flow
FEV1: Forced expiratory volume in 1 second
ECP: Eosinophil cationic protein
PARC: Pulmonary and activation-regulated chemokine
MPO: Myeloperoxidase
AEs: Adverse events

## Background

Proof-of-concept trials with anti-interleukin (IL)5 (reslizumab, mepolizumab), anti-IgE (omalizumab), and anti-IL4 receptor alpha (dupilumab) in chronic rhinosinusitis with nasal polyps (CRSwNPs) recently revealed an innovative therapeutic potential.^1^ These biologicals target specific molecules and immune cells or inflammatory pathways associated with a specific pathomechanism, such as IL-5 orchestrating the survival of eosinophils,^2^ or IL-4 and IL-13 regulating the formation of IgE^3^ and the chemotaxis of eosinophils,^4^, ^5^ among other effects. Beneficial treatment effects such as significantly reduced symptoms and polyp scores, resulting in an increased quality of life, have been seen in patients with severe CRSwNPs, with and without comorbid asthma. Effects of different magnitude and patterns have been illustrated for the different biologics, and they will be discussed here.

Chronic rhinosinusitis (CRS) affects more than 10% of the population in western countries, 12% in the United States;^6^ 11% in the Europe^7^ and 8–11% in Asia.^8^, ^9^ Chronic rhinosinusitis with nasal polyps (CRSwNP), a phenotype of CRS, is a persistent inflammatory disease of the paranasal sinuses, and in 10%–70% accompanied by asthma.^10^ CRSwNP is affecting more than 4% of the population, with about a third of patients not controlled with the current standard of care approach, consisting of topical and systemic glucocorticosteroids (GCS), antibiotics and (often repeated) sinus surgery.^11^ Within 18 months of an endoscopic sinus surgery (ESS), 40% of the CRSwNP patients have been reported with polyp recurrence of disease in three US university clinics.^11^ The physicians then have to deal with the recurrences, and only can offer nasal or oral GCS or repeated surgery, with the likelihood of further recurrence. Based on cluster analysis of the inflammatory mechanisms in patients with CRS, our group for the first time published an approach to endotype CRS, differentiating the disease purely based on biomarkers.^10^ The type 2 clusters largely correlated with phenotypes and furthermore were associated with disease severity, asthma comorbidity and recurrence of diseases. These biomarkers form the targets for innovative therapeutic approaches such as monoclonal antibodies. The first publication is already dated from 2006,^12^ and in the meantime our group has worked with biologics to treat CRSwNP for 12 years. The aim of this review is to summarize the current studies on biologics for nasal polyps, compare the study designs and results of the published data, and further discuss the feasibility of using the biologics as an innovative way to treat CRSwNPs.

## Comparison of biotherapeutics studied in nasal polyposis

Four studies involving 3 monoclonal antibodies (mepolizumab, omalizumab, dupilumab) were included in the comparison. These 3 monoclonal antibodies are blocking different relevant key pathogenic molecules: mepolizumab targets IL-5, omalizumab targets IgE, and dupilumab targets the IL-4 receptor alpha. All studies were RCTs and the articles were published between 2011 and 2018.^13^, ^14^, ^15^, ^16^, ^17^

### Study design

One of the 4 studies was a single center study;^13^ another involved two centers in Belgium,^14^ while the other two were multicenter studies across different countries (Table 1). Sample sizes ranged from 23 to 105 subjects, with treatment groups ranging from 15 to 54, and controls ranging from 8 to 51 participants. The mean age of eligible patients ranges from 47.4 to 51 years with a bilateral nasal polyp score of at least 5 (out of a maximum score of 8) and at least a score of 2 for each nasal cavity despite intranasal corticosteroid treatment. Patients with prior nasal surgery were accepted. Asthma comorbidity is a major characteristic of this study population, being present in 43%–100% of the patients; prespecified enrollment goals were 50% of the patients with comorbid asthma in the dupilumab study and 100% in the omalizumab study. Another important patient characteristic is the history of previous endoscopic nasal surgery, with 58%–100% of the participants reporting former surgery for CRSwNP. In the latest mepolizumab study,^16^ severe recurrent bilateral nasal polyposis was an important inclusion criterion, and all patients had surgery/ies before the study inclusion. However, the number of previous operations was not reported in all studies. As is known, asthma comorbidity and prior nasal surgery may increase the likelihood of a type 2 endotype,^18^ which might be decisive for the response to the biotherapeutics. The dosing and application schemes were tailored to the biologics. In the 2 mepolizumab studies, the dosing regime was identical (750 ​mg intravenous injection every 4 weeks), but the treatment periods were different (8 weeks and 25 weeks), and in the latter study all participants were treated with flixonase aqueous nasal spray throughout the treatment period. The omalizumab and dupilumab studies had the same treatment period of 16 weeks with appropriate dosing regimens (Table 1).

**Table 1:** Study design of RCTs in CRSwNP.

	aMepolizumab^13^	aOmalizumab^14^	aDupilumab^15^	aMepolizumab^16^
Published year	2011^13^	2012^14^	2016^15^	2017^16^
Target molecule	IL-5	IgE	IL-4 receptor alpha	IL-5
Study design*	Single center	Two centers	Multicenter (13 sites)	Multicenter (6 sites)
NO. (verum/placebo)	30 (20/10)	23 (15/8)	60 (30/30)	105 (54/51)
Asthma % (verum/placebo)	43% (50%/30%)	100% (100%/100%)	58% (63%/53%)	78% (81%/75%)
Former surgery % (verum/placebo)	77% (75%/80%)	83% (87%/75%)	58% (63%/53%)	100% (100%/100%)
INCS medication	–	–	MF	FA
Dosing, application	750mg/4weeks × 2 (Intravenous)	According to tIgE levels and body weight, 375 mg max, every 2 weeks or every 4 weeks (subcutaneous)	600/300 mg/week (Subcutaneous)	750mg/4weeks × 6 (Intravenous)
End point and last visit (weeks)	8w/48w	16w/20w	16w/16w	25w/25w

a All these studies were randomized, double-blind, placebo-controlled studies. INCS, intranasal corticosteroid spray; NR, not reported; MF, Mometasone Furoate nasal spray; FA, Flixonase Aqueous nasal spray; tIgE, total serum immunoglobulin E.

### Total nasal endoscopic polyp score

The reduction in total nasal endoscopic polyp score (TPS) was the most important indicator of efficacy. It was assessed as the primary outcome criterion in all compared studies (Table 2). In all 4 studies, the TPS was significantly reduced compared to baseline and also significantly different from controls. The decrease in TPS was most pronounced in the omalizumab study (mean reduction at week 16, verum/placebo ​= ​2.67/0.12). Dupilumab and the latest mepolizumab study had the same mean value of TPS reduction in the treatment group (verum/placebo, 1.9 [95%CI, 1.2 to 2.5]/0.3 [-0.4 to 1.0] for dupilumab at week 16 and 1.9 [SD 0.5]/0.7 [SD 0.5] for mepolizumab at week 25, respectively). In both trials, intranasal corticosteroid spray was applied to all participants, leading to slightly higher mean reductions in TPS in the control groups than the other studies. Considering the treatment differences between mepolizumab and placebo, the mean reduction of TPS in the two mepolizumab studies were similar, although treatment periods (8 weeks and 16 weeks separately) were different.

**Table 2:** Clinical changes from baseline (verum versus placebo).

	aMepolizumab^13^	Omalizumab^14^	Dupilumab^15^	Mepolizumab^16^
TPSs' mean reduction(SD) (verum/placebo)	1.3 (1.72)/0.00 (1.72)，p = 0.028	2.67 (p = 0.001)/0.12 (p = 0.99)	1.9 (1.2–2.5)/0.3 (0.4–1.0) (LS mean, 95% CI), p < 0.001	1.9 (1.4–2.4)/0.7(0.2–1.2) (LS mean, 95%CI)^b^, p ≤ 0.05
Lund-Mackay CT scan score mean reduction (verum/placebo)	Any improvement: 10/2 patients	4.0 (p = 0.02)/-0.5 (p = 0.10)	9.1/0.2 (LS mean) (p < 0.001)	NR
SNOT-22 score reduction (verum-placebo)	NR	NR	18.1 (10.6–25.6) (LS mean, 95%CI), (p < 0.001)	13.2 (4.2–22.2) (LS mean, 95%CI), (p = 0.005)
UPSIT or loss of smell symptom score	Improved (Not significant)	Improved (p = 0.004)	Improved (p < 0.001)	Improved (p < 0.001)
Nasal congestion or obstruction	Improved (Not significant)	Improved (p = 0.002)	Improved (p < 0.001)	Improved (p = 0.002)
Anterior rhinorrhea	No improvement	Improved (p*=* 0.003)	Improved (AM, p < 0.0001; PM, p = 0.0008)	Improved (p < 0.001)
Postnasal drip or mucus in the throat	Improved (not significant)	NR	Improved (AM, p = 0.002)	Improved (p < 0.001)
SF-36	NR	Physical health score Improved (p*=* 0.02)	NR	NR
RSOM-31	NR	Sleep improved (p = 0.03), general symptoms improved (p = 0.01).	NR	NR
AQLQ or ACQ5 reduction	NR	AQLQ Improved (p = 0.003)	ACQ5 Improved (p < 0.001)	NR
PnIF improvement (verum-placebo) L/min	Improved (not significant)	NR	33.1 (12.7–53.5), (LS mean, 95% CI) (p = 0.002)	26.7 (3.1–50.2) (LS mean, 95% CI) (p = 0.027)
FEV1% predicted (verum/placebo)	NR	NR	1.9/9.0, (LS mean) (p = 0.04)	FEV1 (L), not significant

TPS, total nasal endoscopic polyp score; LS, least squares; CI, confidence interval; NR, not reported; SNOT-22, Sino-nasal Outcome Test; UPSIT, University of Pennsylvania Smell Identification Test; SF-36, 36-Item Short Form Survey; RSOM-31, 31-Item Rhinosinusitis Outcome Measuring Instrument; AQLQ, Asthma Quality of Life Questionnaire; ACQ5, 5-item Asthma Control Questionnaire; PnIF, Peak Nasal Inspiratory Flow; FEV1, forced expiratory volume 1. NR, not reported.

a Because of the dropouts, the shown data of this study were all last-observation-carried-forward imputation (LOCF) results.

b The TPSs were conversed from the corresponding figure in published articles by using WebPlotDigitizer v4.1.

The improvement in TPS with omalizumab vs. placebo reached statistical significance from week 8 onwards (after 2 or 4 doses) and in the dupilumab study from week 4 (after 4 doses). Similarly, the mean change from baseline in TPS reached significance for mepolizumab vs. placebo from week 8 (after 2 doses) in the first^13^ and week 13 (after 3 doses) in the latest mepolizumab study.^16^ Patients responding with an improvement in TPS at week 8 were defined as responders (n ​= ​12), accounting for 60% in the first mepolizumab study and stayed statistical significance till week 36 (8 months after the last dose). Other studies did not show long-term data.

### CT score

The changes in TPSs were also reflected in a redaction of polyp burden in the sinuses as measured by CT scan (Table 2). Omalizumab and dupilumab studies showed significant Lund-Mackay score reductions compared with controls (p ​< ​0.001 vs. p ​= ​0.04). Dupilumab (plus mometasone furoate) was more pronounced than with omalizumab (mean reduction, 9.1 vs. 4.0). Unfortunately, the first mepolizumab study only reported that more than half of the mepolizumab-treated patients and less than 20% of placebo group achieved an improvement in CT score compared with baseline,^13^ with no definitive scores, and the latest mepolizumab study did not provide any information of CT scan which resulted in the inability to compare the two biotherapeutics.

### Symptom scores and quality of life measures

Loss of smell, nasal congestion/obstruction, anterior rhinorrhea, and postnasal drip/mucus in the throat are the main symptoms involved in CRSwNP, and the data was generally available in the 4 studies, but the methods used to assess them were different. The effect of dupilumab on olfactory improvement was assessed by UPSIT (an objective measurement ranging from 0 to 40, higher scores of 35–40 indicate normal sense of smell), while the other 3 symptoms were captured using a categorical scale (0 ​= ​no symptoms, 1 ​= ​mild symptoms, 2 ​= ​moderate symptoms, and 3 ​= ​sever symptoms). The categorical scale was used to assess all 4 symptoms in the first mepolizumab study and 3 symptoms (postnasal drip was not reported) in the omalizumab study. However, the latest mepolizumab study used a VAS scoring method and the results were presented in centimeters (range 0–10). The variation in the scoring methods hindered further comparison between different biologics. Therefore, the effectiveness in symptom improvements of the monoclonal antibodies can only be judged from the treatment difference in a single study (Table 2). Significant improvements in nasal symptoms were documented in all three studies (omalizumab, dupilumab, the latest mepolizumab) except the first mepolizumab study. The first mepolizum study has shown that the nasal symptoms were improved but did not reach significance although the TPS was significantly reduced.^13^ Remarkably, the improvement in loss of smell in this study was reported to stay at the same level during the whole period of follow-up (11 months after the last dose), whereas the other symptoms normalized after a period of time.

Quality of life scores are important clinical indicators for assessing disease severity and are also important in the evaluation of the efficacy of biotherapeutics. Different scoring methods were designed in the four studies (SNOT-22, UPISIT, SF-36, RSOM-31, AQLQ, ACQ5, et al.). Two studies provided the SNOT-22 scores,^15^, ^16^ and according to the outcome, dupilumab was more effective than mepolizumab in reducing the SNOT-22 total score (LS mean reduction, 18.1 [95% CI 10.6 to 25.6] vs 13.2 [95% CI 4.2 to 22.2]).

### Nasal airflow (PnIF) and lung function (FEV1)

Additionally to symptom scores, PnIF and FEV1 are undoubtedly objective clinical indicators. Dupilumab was reported to be effective in improving PnIF and FEV1, and was superior to mepolizumab which did not reach statistical significance in improving FEV1. Mepolizumab^13^ did not yield a statistically significant benefit concerning the PnIF volume (p ​= ​0.10), which was in contrast to subsequent research, possibly due to the different treatment duration of 8 vs. 25 weeks.

### Biomarkers

Patients with CRSwNP with comorbid asthma and/or former surgeries with disease recurrence likely express type 2 inflammation.^10^, ^19^ The currently developed biologics based on type 2 cytokines and their downstream products, including anti-IL-5, anti-IL-4, anti-IL-13 and anti-IgE, are targeted at CRSwNP patients. Other cytokines like IL-33 and thymic stromal lymphopoietin are also produced in patients with CRSwNP^19^ and are potential therapeutic targets for this disease. However, no biomarkers are currently known to select between these possible strategies. No difference in the baseline characteristics was found in the responders vs. the non-responders in the mepolizumab-treated group. In particular, no difference was found for IL-5 levels and blood eosinophil counts. Omalizumab worked comparably in non-allergic compared with allergic CRSwNP patients. Mepolizumab, blocking IL-5, reduced blood eosinophil count, ECP and IL-5Rα in serum and several biomarkers (IL-1β, IL-5Rα, IL-6 and MPO) in nasal secretions significantly.^13^, ^16^ Omalizumab, blocking IgE by complexing the molecules and reducing free IgE, didn't induce any significant changes in the serum and nasal secretion parameters by treatment.^14^ In CRSwNP patients, tissue IgE levels were reported to relate to the severity of disease, the presence of comorbid asthma, and the presence of IgE to *Staphylococcus aureus* enterotoxins, while being independent of total serum IgE levels or allergy skin prick test responses.^20^, ^21^, ^22^ Until now, only in the dupilumab study data on biomarkers in nasal polyp tissue was reported.^17^ Dupilumab reduced tissue ECP, pulmonary and activation-regulated chemokine (PARC) and eotaxin-1 significantly compared with placebo in polyp tissue, total IgE and Eotaxin-3 in nasal secretions, and plasma eotaxin-3, serum total IgE and eosinophils in the circulation.^15^, ^17^ This demonstrated that dupilumab targets a spectrum of type-2 inflammatory markers at organ and systemic levels (Table 3).

**Table 3:** Comparison of mean changes from baseline in biomarkers between treatment and control groups.

		aMepolizumab^13^	Omalizumab^14^	bDupilumab^15^^,^^17^	Mepolizumab^16^
Blood Eosinophils		Reduced (p < 0.001)	NR	Not significant (Temporary increase in some patients)	Reduced (Baseline mean 500 cells/ml drop to 50 cells/ml EoT)
Plasma eotaxin-3		NR	NR	Reduced (p < 0.001)	NR
Serum	Total IgE	NR	Complex	Reduced (p < 0.001)	NR
	ECP	Reduced (p = 0.022)	NR	NR	NR
	IL-5Rα	Reduced (p < 0.001)	NR	NR	NR
	TARC	NR	NR	Not significant	NR
Nasal Secretions	Total IgE	Not significant	NR	Reduced (p < 0.05)	NR
	ECP	Not significant	NR	Not significant	NR
	IL-1β	Reduced (p = 0.043)	NR	NR	NR
	IL-5	Not significant	NR	NR	NR
	IL-5Rα	Reduced (p = 0.010)	NR	NR	NR
	IL-6	Reduced (p = 0.020)	NR	NR	NR
	MPO	Reduced (p = 0.009)	NR	NR	NR
	Eotaxin-3	NR	NR	Reduced (p < 0.001)	NR
Nasal polyp tissue	Total IgE	NR	NR	Reduced (not significant)	NR
	IL-13	NR	NR	Reduced (not significant)	NR
	ECP	NR	NR	Reduced (p < 0.01)	NR
	PARC	NR	NR	Reduced (p < 0.01)	NR
	Eotaxin-1	NR	NR	Reduced (p < 0.05) (Not significant from baseline)	NR
	Eotaxin-2	NR	NR	Reduced (not significant)	NR
	Eotaxin-3	NR	NR	Reduced (not significant)	NR

ECP, eosinophil cationic protein; NR, non reported; IL, interleukin; IL-5R α, IL-5 receptor α subunit; TARC，Thymus and Activation-Regulated Chemokine; PARK, pulmonary and activation-regulated chemokine; IL-1β, IL-1β subunit; MPO, myeloperoxidase. NR, not reported.

a Because of the dropouts, the shown data of this study were all last-observation-carried-forward imputation (LOCF) results.

b The nasal polyp biopsies were collected with 12 patients at baseline and Week 16 (n = 4 placebo, n = 8 dupilumab). Ref ^17^ covers the nasal biomarker data of the clinical study presented in ref ^15^.

#### Safety and adverse events

A variety of adverse events (AEs) were reported by the 4 RCTs, and most of them were expected, with the most common AEs related to upper airway infections (common cold, nasopharyngitis and headache). Eight serious adverse events were reported in total, of which 3 patients in the treatment group (1 patient treated with mepolizumab reported diverticulitis, 2 patients treated with dupilumab reported herpes zoster, arrhythmia and upper extremity pain or numbness). However, none of the reported serious adverse events were deemed to relate to the biotherapeutics (Table 4). There were 4 reported serious adverse events in the control group of the dupilumab study.

**Table 4:** Adverse events reported in the biologics studies.

	Mepolizumab^13^	Omalizumab^14^	Dupilumab^15^	Mepolizumab^16^
Total number of adverse events No. (%) of patients, verum/placebo	Events, 21/3 N (%) of all patients, 16 (53%)	Events, 24/8	30 (100%)/25 (83%)	40 (75%)/42 (81%)
Most frequent adverse events % Of the patients, verum/placebo	Common cold (25%/10%) Bronchitis (15%/0%) Sinusitis (10%/10%) Short of breath (5%/0%) Headache (5%/0%)	Common cold (53%/0%) Frontal headache (27%/13%) Nasal obstruction (20%/20%) Shortness of breath (13%/13%) Otitis media (13%/0%)	Nasopharyngitis (47%/33%) Injection-site reaction (40%/7%) Oropharyngeal pain (23%/7%) Epistaxis (23%/7%) Headache (20%/17%)	Headache (25%/38%) Nasopharyngitis (19%/23%) Oropharyngeal pain (11%/8%) Back pain (9%/0%) Influenza (8%/4%)
Serious adverse events, verum/placebo	a1/0	None	a3**/**4	None

a The serious adverse event was not considered to be related to the study drug.

## Discussion

Chronic Rhinosinusitis with nasal polyps (CRSwNP) is an often severe persistent airway disease, which in Europe and the US is mainly characterized by a type-2 inflammatory reaction. In spite of current treatment recommendations such as topical GCS twice daily, recurrences after oral GCS or surgeries are frequently observed. About one-third of the patients with CRSwNP are considered control failures with the current treatment options. This indicates a clear unmet need to search for other treatment possibilities, which need to be structured in adequate diagnosis and treatment care pathways.^23^

It is possible that better surgical techniques such as the “reboot technique”, aiming to remove all inflamed mucosa of the sinuses, but leaving nasal mucosa untouched, would change this situation.^24^ The nasal epithelium covers the sinuses after just 4–6 weeks and forms a smooth ciliated epithelial barrier with goblet cells. No additional complications have been observed with this technique in comparison to the current standard, but significantly reduced recurrence rates could be demonstrated over 30 months post-operatively. However, this technique is time demanding and asks special skills from the surgeon, and it has not found broad acceptance yet. Furthermore, type 2 inflammatory nasal polyps are frequently characterized by asthma comorbidity, which will not be controlled by surgery.

Our group, therefore, have performed several proof-of-concept studies in Ghent since 2005, starting with several investigator-initiated studies; we demonstrated the effect of a monoclonal antibody to IL-5, reslizumab, on the polyp score and symptoms, showing that in principle biologics could work in CRSwNP.^12^ The hypothesis on the role of this antibody was derived from a former study in 1997 showing that IL-5 protein is increased in nasal polyps and that this cytokine correlates to eosinophil presence.^25^ We also showed in the same year that nasal polyp eosinophils in tissue have a longer survival time compared to blood eosinophils and that anti-IL-5, but not anti-IL-3 or anti-GM-CSF could induce apoptosis in tissue eosinophils,^26^ being confirmed in human disease today. Further studies on the regulation of the IL-5 receptor on eosinophils, describing transmembrane and soluble receptors in polyps,^27^ will help to appreciate treatment effects of benralizumab, an IL-5 receptor antagonist currently in Phase 3 trials.

Work on IgE in nasal polyps, specifically on *Staphylococcus aureus* enterotoxin-specific IgE (SE-IgE) pointed to the role of IgE^28^; in the meanwhile it was demonstrated that anti-IgE (omalizumab) can successfully be used in allergic as well as non-allergic type-2 inflammatory polyps.^14^ Finally, in a cluster analysis of biomarkers in CRS enabling an endotyping approach to CRS,^10^ we demonstrated the differentiation of CRSwNP in non-type 2, moderate and severe type 2 subgroups. A first trial with dupilumab, an anti-IL-4 receptor alpha antagonist blocking IL-4 and IL-13 activities,^15^ showed that a broader impact on type 2 immune reactions does lead to the reduction of IgE as well as of local mucosal eosinophil migration from the blood and activation,^17^ resulting in an even more pronounced effect on nasal polyps, smell, quality of life and asthma comorbidity.

The studies discussed here are proof-of-concept trials with relatively small patient numbers and limited observation time, largely based on the nasal polyp score (TPS); the TPS is stable over time under placebo treatment—in clear contract to symptom scores or quality of life questionnaires, showing placebo effects – and allows the differentiation of verum vs. placebo with small subject numbers. Interestingly, all biologics result in a reduction of the TPS by about 2 score points, representing a clinical meaningful reduction equal to a two weeks oral GCS course. Still, small differences in the results suggest a small superior effect for dupilumab with respect to asthma control, smell, CT scan scores and SNOT-22 questionnaire.^15^

As demonstrated, CRSwNP in approximately 85% represents a type 2 inflammation (10); additionally, in these studies, 43–100% of the patients suffered from comorbid asthma, 58–100% had at least one former surgery, and all patients had a polyp score of minimum 5 out of 8. Thus, patients included into these studies showed type 2 inflammation in probably 90–100% of subjects. As a consequence, serum IgE and blood eosinophil counts were above normal limits for most patients, but not all, in the studies in which those parameters were monitored. However, we did not select patients based on biomarkers, as no biomarkers have been established for individual CRSwNP patients, and no restrictions in the application of the monoclonal antibodies are identified so far. However, patients not showing a type 2 immune reaction are expected not to respond to the treatment.

In evaluating these small studies, however, one has to appreciate that the populations were different in terms of asthma comorbidity, probably also in severity of type 2 inflammation, and treatment duration. Therefore, we need to await Phase 3 studies, currently run and evaluated, to decide on evidence-based care pathways, selection criteria as well as stopping rules.^18^ Also in terms of safety, although biologics were well tolerated in the small studies, more robust data will be available with the Phase 3 trials being published (see Box).

Box **Remaining questions**

Which cells and mediators are crucial to target in order to optimize the approach with biologics?

Are there subgroups within the type 2 positive subjects, asking for a differentiation of therapy with different biologics?

Which clinical and biological markers would help to select the right therapy for a given patient?

Which stopping rules apply for the different biologics in order to avoid ineffective treatment?

What will be the role of biologics in care pathways for CRSwNP treatment, specifically in comparison to systemic GCS and surgery?

Alt-text: Box
